# Dating Applications, Sexual Behaviors, and Attitudes of College Students in Brazil’s Legal Amazon

**DOI:** 10.3390/ijerph17207494

**Published:** 2020-10-15

**Authors:** Maycon Klerystton B. Tavares, Romulo L. P. de Melo, Bianca F. da Rocha, Débora J. Andrade, Danielle R. Evangelista, Márcia C. T. S. Peres, Leonardo R. Baldaçara, Thiago DeSouza-Vieira, Elisangela V. Assis, José Bruno N. F. Silva

**Affiliations:** 1 Medicine Course, Universidade Federal do Tocantins, 77001-923 Palmas, Tocantins, Brazil; mayconklerystton@gmail.com (M.K.B.T.); biancarocha7@mail.uft.edu.br (B.F.d.R.); deborajandrade@mail.uft.edu.br (D.J.A.); marciaperes@mail.uft.edu.br (M.C.T.S.P.); leonardobaldassara@gmail.com (L.R.B.); 2 Psychology Course, Faculdade Santa Maria, 589000-000 Cajazeiras, Paraíba, Brazil; romulo.psiq@gmail.com; 3 Nurse Course, Universidade Federal do Tocantins, 77001-923 Palmas, Brazil; daniellerosa@mail.uft.edu.br; 4 Vector Molecular Biology Section, Laboratory of Malaria and Vector Research, National Institutes of Allergy and Infectious Diseases, National Institutes of Health, Rockville, MD 20850, USA; thiago.soaresdesouzavieira@nih.gov; 5 Medicine Course, Universidade Federal de Campina Grande, 58900-000 Cajazeiras, Paraíba, Brazil; elisangela.vilar@ufcg.edu.br

**Keywords:** dating apps, sexual behaviors, attitude, college students, sexual health

## Abstract

Although dating applications (apps) have become popular among young adults, there is a dearth of information regarding the sexual health implications among Brazilian college students. This study examined risky sexual behavior and attitudes of dating app users, based on their sex in Brazil’s Legal Amazon. Three hundred and fifty-nine students reported their sociodemographic data, dating app use, and sexual behaviors and attitudes through self-administered questionnaires. Bivariate analyses and analysis of variance (ANOVA) with Bonferroni post-hoc tests were performed. Dating app use was reported by 238 (66.3%) subjects, most of whom had an encounter and sex with a casual partner. Women frequently requested condom use. Trust in one’s partner or having repeated encounters were the main reasons for engaging in risky sexual behavior. Men had a greater number of sexual partners and less protective attitudes. Sexual health awareness by apps was not reported by 97% of women, and most of them were not tested for sexually transmitted infections. A positive attitude toward sexual health was not a predictor of safe sex. Important similarities and differences regarding risky sexual behaviors and attitudes were observed between the sexes, many of which correlated with increased sexual vulnerability during the sexual encounters arranged through the dating apps. This cross-sectional study supports efforts on sexual health promotion and sexual education implementation in the face of growing usage of apps among young adults for sexual matters.

## 1. Introduction

Technology has become an essential tool for education and social communication [1]. The advent of the Internet boosted interpersonal relationships, influenced by virtual rooms and visual and digital mobile communication. Students use educational technology as a means of transmitting knowledge, and mobile technology plays a role in the interactions of this population through rapid conversation feedback, social support, well-being, and encounters [2,3].

Dating applications (apps) have become a part of many people’s lives. Most apps are free, and users can chat with a large number of people through location-based algorithms using a Global Positioning System (GPS). Interpersonal encounters arranged through apps vary from social networking to finding sexual partners [4,5,6,7].

Sexual behavior includes all activities in which individuals explore their sexual life and sexuality across cultural influences, biological elements, and social groups, involving solitary or partnered sex, relationships, reproductive health, contraception, and sexually transmitted infections (STIs) [8]. Previous studies have reported negative sexual health implications for dating app users. Drug use, compulsivity, deception, risky behaviors [9], sexual abuse [10], attitudes [11] associated with socio-sexuality, and previous reports of STIs [12] are more prevalent in this population. Although factors predicting engagement in casual sex for both sexes from the use of dating apps [13] and risky sexual behaviors and attitudes among young adults, college students and men who have sex with men (MSM) [14,15,16] have been thoroughly addressed, there has been little emphasis on biological sex, beyond sexual orientation [17].

In Brazil, the National School-based Health Survey studies have reported that the first experience with sexual intercourse usually occurs during adolescence at an average age of 13 years [18,19,20]. Although data on the prevalence of STIs among Brazilian adolescents are limited, early sexual initiation is associated with unprotected sex, which may contribute to the high prevalence of STIs and unplanned pregnancy among Brazilian young adults [21,22]. Theme-Filha et al. [23] found that, in Brazil, more than 50% of all pregnancies carried to term were unplanned and were associated with younger age, brown and yellow skin color, substance use, and lower education level. Students report not having received pregnancy prevention counseling or sexual health education [24]. Birth control methods reported by women of reproductive age include oral and/or injectable contraceptives, tubal ligation, and male condoms [25,26,27]. In addition, multiple sexual partners and non-adherence to prophylactic measures after exposure to risk are the factors commonly reported by young Brazilian adults and college students [22,28], which differ between men and women [21,29].

The existing research examines risky sexual behavior of Brazilian dating app users primarily in the context of sexual orientation, more specifically for single, young MSM with high education levels. The most commonly reported dating apps for sex-seeking in this population are Grindr, Hornet, Scruff, and Tinder [30,31,32]. Recently, Lopes and Vogel [33] found that Brazilian women use Tinder for other social interactions, such as looking for people, friends, casual dates, and romance.

Notably, youth is an important time for social development and may be a critical period for promoting healthy behaviors and attitudes that will help protect young people from issues like STIs. The large number of young adults in the academic environment makes this an ideal space for studying factors related to the digital era, since access to apps and casual short-term relationships are common in this population. Investigating the association between the use of dating apps and the sexual health of Brazilian college students is essential for guiding decision-making and interventions related to the quality of life for these students. Therefore, the objective of this study was to analyze risky sexual behavior and attitudes of dating app users, based on their sex, at a public university located in Palmas, Tocantins, in Brazil’s Legal Amazon.

## 2. Materials and Methods

### 2.1. Design

This was a cross-sectional study examining the sexual behaviors and attitudes of college students. Data were collected from August 2018 to April 2019. A self-administered, anonymous questionnaire was employed to obtain the variables of interest. The ballot-box technique was used to ensure the confidentiality of the responses, to improve reliability, and to avoid social desirability bias. The participants were informed that they could skip questions they did not want to answer. Details about the participants, procedures, measurements, and statistical analysis are provided below.

### 2.2. Participants

A convenience sample of college students was recruited from the Federal University of Tocantins, Palmas, Brazil. A recruitment booth was set up in common areas of the university campus. Thus, a total of 465 men and women aged ≥18 years were invited. Fifty-three (11.39%) chose not to participate in the study. A sample of 412 answered the questionnaire. Fifty-three questionnaires (12.86%) were excluded due to missing data for more than half of the answers for age, sex, or questions related to the use of dating apps. The final sample consisted of 359 students.

### 2.3. Procedures

The study protocol was approved by the research ethics committee of the Federal University of Tocantins (No. 2758178). The participants voluntarily signed the Informed Consent Form after being informed about the objectives of the study. No compensation was offered to participate in this study.

### 2.4. Measurements

The participants were asked about their age, sex, race/ethnicity, family income, smoking habits, alcohol consumption, sexual orientation, marital status, and relationship status. Further, the participants were categorized into three groups based on their age: adolescents (18–19 years), young adults (20–24 years), and adults (>24 years) [34]. They indicated whether they used/have used dating apps and whether they had casual encounters with a partner they met through the app [4,10].

The sexual behaviors of app users were evaluated using the following questions, based on previous studies [4,5,16]. Have you had sexual intercourse during a casual encounter (yes/no)? Have you used protection during sexual intercourse (yes/no)? Reasons for engaging in safe sex: (i) I had a condom; (ii) my partner asked me to use a condom; (iii) I asked my partner to use a condom. Reasons for engaging in unsafe sex: (i) I asked, but my partner did not use a condom; (ii) we did not have a condom; (iii) trust in my partner/previous encounters. How many sexual partners have you had in the past three months? Have you been tested for an STI after intercourse? Was the test result positive? Have you received information about safe sex/STIs through the dating apps?

Positive attitude toward sexual health was assessed using seven items on a three-point Likert scale (0 = not important; 1 = important; 2 = very important). Topics included health promotion policies at the university, reflecting on safe sex before finding a casual partner, using a condom during sexual intercourses with a continuous partner, consulting a specialist for information on safe sex/STIs/testing, talking about STIs with your partner, knowing the sexual history and health status of your partner, and refusing unsafe sexual intercourse.

### 2.5. Statistical Analysis

The data were analyzed using descriptive statistics. Categorical variables were expressed as relative and absolute frequencies. The participants were divided into two groups: men and women. First, to explore the association of biological sex with the key variables of interest, Chi-square and Fisher’s exact tests were performed. Relationships were examined between age group, sexual orientation, family income, race/ethnicity, marital status, relationship status, smoking habit, alcohol consumption, and use of dating apps for both women and men. In addition, the same tests were used to determine the associations between sex and categorical sexual behavior variables, including the reasons for safe and unsafe sex. Then, a factorial one-way analysis of variance (ANOVA) with a Bonferroni post-hoc test was used to understand sex differences in positive attitude, app use, and sexual practice. In this case, sex, use of dating apps, casual partner encounters, sexual practice, and safe sex were categorized as independent variables, and the sum of all attitude values was considered the dependent variable. Measures of central tendency (mean) and dispersion (standard deviation) were used. The data were analyzed using SPSS Statistics software version 25 (IBM Corp., Armonk, NY, USA), with a significance level of *p* ≤ 0.05, with a 95% confidence interval.

## 3. Results

As shown in Table 1, of the 359 participants, 186 (51.8%) were men and 173 (48.2%) were women. There was no statistically significant difference between the sexes in age, family income, color/race, marital status, smoking habit, or alcohol consumption. The median age was 21 years. The participants were predominantly heterosexuals, but the number of homosexuals was higher in the male group (*p* < 0.05), as was the frequency of casual encounters (*p* = 0.043) (Table 1).

To investigate sexual behaviors, 238 (66.3%) participants who reported the use of apps (*p* = 0.002) were included (Table 1). There was no statistically significant difference between sex and casual encounters. However, 82.9% of men reported sexual intercourse in casual encounters (*p* = 0.058). Men and women reported similar rates of condom use. Unsafe sex was mostly reported by young adults (*p* = 0.014) and single men with casual encounters (*p* = 0.039), regardless of sex (Table 2).

Most women requested the use of a condom. Among men, 49.2% had a condom and 15.3% received requests to use it (*p* < 0.05). Not having a condom or trust/repeated encounters were the most frequent reasons for unsafe sex. The reasons for using or not using a condom were not reported by five participants. The frequency of having multiple sexual partners in the previous three months was higher for men (*p* = 0.024). Among dating app users, women did not receive sexual health information through the app (*p* < 0.01). There was no statistically significant difference between the sexes for being tested after intercourse. Overall, 54.3% did not undergo serology tests (*p* = 0.095) (Table 2). However, the majority of those who underwent serology testing received health promotion information (*p* < 0.01). In addition, two men and two women reported testing positive for an STI (6.3%).

The one-way ANOVA followed by the Bonferroni test showed a statistically significant interaction between the use of dating apps and a protective attitude toward sexual intercourse (F (1; 348) = 3.70; *p* = 0.05). Men who used dating apps had a lower mean protective attitude score than those who did not use dating apps. By contrast, female dating app users had a higher mean protective attitude score (Table 3).

The interaction phenomenon was not statistically significant for participants who had encounters through dating apps (F (1; 235) = 1.25; *p* = 0.26) or sexual intercourse with a partner (F (1; 174) = 0.09; *p* = 0.76). However, the highest mean positive attitude was found in women who had sexual intercourse (simple effect; F (1; 235) = 25.53; *p* < 0.001). Finally, there was a statistically significant effect for the interaction between biological sex and safe sex practice during an encounter (F (1; 135) = 4.21; *p* = 0.04) (Table 3). Protective attitudes toward sexual intercourse were lower in men who had unsafe sex or underused condoms. However, even in situations where one was vulnerable to unsafe sex, women had a higher mean score for attitudes toward sexual care (Table 3).

## 4. Discussion

In this study, most dating app users reported having sex with the casual partners they met through the app. These data corroborate those of Choi et al. [4] and Lehmiller et al. [12]. Using dating apps allows for more chances to talk and to find a potential sexual partner, while preserving distance and reducing the risk of embarrassment in case of a declined invitation. This study showed that at least one in two sexually active college students reported not using or underusing condoms. Young adult participants and those reporting casual encounters were more likely to engage in this risky behavior. There is evidence that men and women use online resources to increase sexual activity [35], that there is low condom use among young or college-aged app users [4], and that participants in casual relationships are more likely to engage in unsafe sex [36].

Data from the current study showed that, compared to men, women are less likely to have a condom available. Thus, even with sexual health policies that encourage the use of female condoms, women’s adherence to this contraceptive method is still low. One possible explanation is that in sociocultural relationships, carrying a condom is considered necessary for sexual activities, but the behavior is often attributed to men by hegemonic male role norms [37]. Thus, these findings indicate that patriarchal relationships may persist in Brazilian society, inferring that men have power over women’s sexuality, which limits sexual freedom for women [38].

Although a partner’s refusal to use a condom during sexual intercourse is associated with sex inequality and social factors [39,40], this study found no differences between the sexes. Sexual coercion was reported by 8.5% of the participants. One concerning factor is that these apps could be used by sexual abusers, particularly because app users were more likely to find a partner who would insist on sex without a condom [10].

Trust in one’s partner and repeated encounters were the main reasons for engaging in unsafe sex. Neglecting sexual protection is influenced by bonds of intimacy and trust [37]. Thus, some studies have reported that trust is equally influenced by both biological sex and gender, and it can be an essential factor in regulating condom use [41,42]. In addition, having a stable sexual partner results in decreased condom use, especially in young adults [43]. However, in our sample, exposure to multiple sexual partners was reported three times more by male participants. Some studies have reported the influence of gender on sexual practice, showing that men tend to seek multiple sexual partners using apps and websites [6,44].

Most female dating app users received no sexual health information. Huang et al. [45] reported a small number of apps with health promotion policies, some of which focused on MSM. Safe sexual health information on dating apps can be an intervention method to reduce risky sexual behavior. This is justified because participants who investigated STIs searched for health guidance via dating apps. Thus, most participants were never tested for STIs after intercourse with a casual partner. These data corroborate a study by James et al. [46] in which half of the sexually active college students had never been tested for HIV. One possibility for increasing testing in this population is to provide testing at counseling centers, ensuring support since the person looks for the service until referral to clinical care.

A positive attitude toward sexual health was inverted for women. Knowledge, tradition, beliefs, and family values influence decision-making regarding sexual practice [47]. In this study, a healthy sexual practice attitude, although higher among women, was not a predictor of safe sex. This phenomenon corroborates the study by Widman et al. [48], who reported low adherence to condom use even in women with a positive attitude toward health promotion.

Although a positive attitude increases good behavior, the implementation of preventive measures, the search for knowledge, and conversations with one’s partner may be affected, especially if vulnerability factors favor this behavior change [49]. In this context, the Internet can affect women’s decisions to engage in risky sexual behaviors and obtain specific sexual health information [50]. Another hypothesis is that, at the time of sex, although women recognize its importance, their willingness to use protection is undermined by men. This theory has important consequences for the influence of sex education. Men need to be educated to recognize the importance of safe sex and to respect their partner’s desire for protection, and women need to be educated to recognize the importance of protection and to ensure that their desires are respected. In this study, men who had unsafe sex saw healthy sexual conduct as less important. Studies that report the emotional and psychological vulnerability of app users to risky attitudes and behaviors are necessary to better understand the practice of unsafe sex.

This study has some limitations. First, this is an analytical study with a cross-sectional design, preventing the differentiation of casual as opposed to stable relationships. Second, the data represent participants recruited by convenience sampling on a university campus. However, the results were similar to those of other studies involving university app users. Third, the risky sexual behavior of students who reported not using this type of technology was not analyzed. Future studies are necessary to understand the behavior of this population in order to increase the implementation of health promotion campaigns in the academic field. Although the questionnaire was self-administered, which may have a recall bias, this type of methodology is common in studies on behavioral health and attitude. In addition, although a five-point Likert scale is more accurate, we used a three-point scale to assess positive attitudes toward sexual health in order to limit the complexity of the decision-making process, consequently decreasing the time for the participant to answer the questionnaire. Although the study has limitations, this was the first Brazilian study to evaluate the prevalence of dating apps among Brazilian university students and investigate aspects of sexuality and biological sex in this population.

## 5. Conclusions

This study found risky sexual behaviors in college-aged app users. Unsafe sex was present in both biological sexes, even with a positive attitude toward sexual health reported by women. Men had a greater number of sexual partners and less protective attitudes. Most students were not tested for STIs after intercourse with a casual partner. Further studies on sex education and on the influence of other factors are needed to understand condom underuse and to increase sexual protection. The provision of sexual health counseling via apps and universities should be consistent with safe sex practices.

## Figures and Tables

**Table 1:** Bivariate analysis of the sociodemographic characteristics of students at the Federal University of Tocantins, Palmas campus (*n* = 359), *n* (%).

Variables		Sex		*p*
		Male (*n* = 186)	Female (*n* = 173)	
Age group	18–19	58 (31.2)	55 (31.8)	0.529
	20–24	104 (57.5)	92 (53.2)	
	>24	21 (11.3)	26 (15)	
Sexual orientation	Heterosexual	127 (68.3)	140 (80.9)	<0.001
	Homosexual	33 (17.7)	06 (3.5)	
	Bisexual	26 (14)	27 (15.6)	
Family income	Up to 3 salaries	77 (41.4)	64 (37)	0.393
	>3 salaries	109 (58.6)	109 (63)	
Race/ethnicity	Yellow	2 (1.1)	2 (1.2)	0.991 (*)
	White	56 (30.1)	49 (28.3)	
	Pardo	93 (50)	89 (51.4)	
	Black	33 (17.7)	31 (17.9)	
	Not declared	2 (1.1)	2 (1.2)	
Marital status	Single	179 (96.2)	159 (91.9)	0.159 (*)
	Married	3 (1.6)	8 (4.6)	
	Divorced	-	2 (1.2)	
	Stable union	4 (2.2)	4 (2.3)	
Relationship status	Currently in a relationship	58 (31.4)	71 (41.3)	0.043
	Casual encounters	65 (35.1)	41 (23.8)	
	Not dating	62 (33.5)	60 (34.9)	
Smoking habit	No	145 (79.2)	147 (85)	0.159
	Yes	38 (20.8)	26 (15)	
Alcohol consumption	No	68 (36.6)	76 (43.9)	0.154
	Yes	118 (63.4)	97 (56.1)	
Use of dating apps	No	49 (26.3)	72 (41.6)	0.002
	Yes	137 (73.7)	101 (58.4)	

* Fisher’s exact test.

**Table 2:** Factors associated with casual partner encounters and sexual behaviors by sex, *n* (%).

Variables		Sex		*p*
		Male (*n* = 137)	Female (*n* = 101)	
Have you ever had a date through the app? (*n* = 238)	No	32 (23.4)	29 (28.7)	0.350
	Yes	105 (76.6)	72 (71.3)	
Have you received information about safe sex/STI through apps? (*n* = 238)	No	97 (70.8)	98 (97)	<0.001
	Yes	40 (29.2)	3 (3)	
Have you had sex? (*n* = 177)	No	18 (17.1)	21 (29.2)	0.058
	Yes	87 (82.9)	51 (70.8)	
Have you had safe sex? (*n* = 138)	No/Sometimes	28 (32.2)	19 (37.3)	0.544
	Yes	59 (67.8)	32 (62.7)	
Multiple sexual partners	No	48 (55.2)	38 (74.5)	0.024
	Yes	39 (44.8)	13 (25.5)	
STI testing	No	42 (48.8)	33 (63.5)	0.095
	Yes	44 (51.2)	19 (36.5)	
Reasons for condom use during sexual intercourse with casual partner (*n* = 90)				
I had my condom		29 (49.2)	2 (6.5)	<0.001
My partner requested the use		9 (15.3)	7 (22.6)	
I requested the use		21 (35.6)	22 (71)	
Reasons for unsafe sexual intercourse with casual partner (*n* = 43)				
I asked, but my partner did not use a condom		2 (7.6)	2 (12.5)	0.577 (*)
We did not have a condom		12 (46.2)	5 (31.3)	
Trust in the partner/previous encounters		12 (46.2)	9 (56.2)	

* Fisher’s exact test.

**Table 3:** Analysis of attitude toward sexual health and use of dating applications, casual partner encounter, sex practice, and safe sex.

	Variables		Use of Dating Apps *		Casual Partner Encounters		Sexual Practice		Safe Sex ^#^	
			Mean (SD)	CI 95%	Mean (SD)	CI 95%	Mean (SD)	CI 95%	Mean (SD)	CI 95%
Sex	Male	No	11.44 ^a^ (1.81)	10.84–12.04	10.78 ^a^ (2.07)	10.03–11.53	11.17 ^a^ (0.52)	10.13–12.20	10.00 ^a^ (2.70)	9.12–10.87
		Yes	10.69 ^b^ (2.27)	10.34–11.03	10.65 ^a^ (2.34)	10.97–13.54	10.55 ^a^ (0.24)	10.07–11.01	10.81 ^a^ (2.21)	10.20–11.41
	Female	No	12.00 ^a^ (1.87)	11.51–12.49	12.76 ^a^ (1.64)	11.97–13.54	12.52 ^a^ (0.48)	11.57–13.48	12.26 ^a^ (1.85)	11.20–13.32
		Yes	12.16 ^a^ (1.96)	11.75–12.57	11.91 ^b^ (2.96)	11.41–12.41	11.66 ^a^ (0.31)	11.04–12.28	11.29 ^a^ (2.48)	10.46–12.12

* Effect of the interaction between biological sex and use of applications and sexual attitude (*p* = 0.05); ^#^ Effect of the interaction between biological sex and safe sex (*p* = 0.04). Different letters in the means represent statistically significant differences (*p* ≤ 0.05) within the sexes.
